# Supplementary material for: A fault-intrusion-tolerant system and deadline-aware algorithm for scheduling scientific workflow in the cloud
Source: PeerJ Comput Sci. 2021 Nov 2;7:e747. doi: 10.7717/peerj-cs.747 (PMC8576554; doi:10.7717/peerj-cs.747)
Supplement: Supplemental Information 6 [file peerj-cs-07-747-s006.zip › Myproject1.1/docs/index-files/index-8.html]

H-Index


JavaScript is disabled on your browser.


- Overview
- Package
- Class
- Use
- Tree
- Deprecated
- Index
- Help

- Prev Letter
- Next Letter

- Frames
- No Frames

- All Classes

A B C D E F G H I J L M N O P Q R S T U V W Z 


## H

HarddriveStorage - Class in org.cloudbus.cloudsim
:   An implementation of a storage system.

HarddriveStorage(String, double) - Constructor for class org.cloudbus.cloudsim.HarddriveStorage
:   Creates a new harddrive storage with a given name and capacity.

HarddriveStorage(double) - Constructor for class org.cloudbus.cloudsim.HarddriveStorage
:   Creates a new harddrive storage with a given capacity.

hasChecked - Variable in class org.workflowsim.clustering.AbstractArrayList
:   A check point.

hasChecked - Variable in class org.workflowsim.clustering.TaskSet
:   the check point.

hasLocalFile(File) - Method in class org.workflowsim.CondorVM
:   Tells whether a file is in the local file system

hasPotentialAvailableSpace(int) - Method in class org.cloudbus.cloudsim.HarddriveStorage
:   Checks whether there is enough space on the storage for a certain file.

hasPotentialAvailableSpace(int) - Method in interface org.cloudbus.cloudsim.Storage
:   Checks whether there is enough space on the storage for a certain file.

hasReserved() - Method in class org.cloudbus.cloudsim.Cloudlet
:   Checks whether this Cloudlet is submitted by reserving or not.

hasReserved() - Method in class org.cloudbus.cloudsim.ResCloudlet
:   Checks whether this Cloudlet is submitted by reserving or not.

Helper - Class in org.cloudbus.cloudsim.examples.power
:   The Class Helper.

Helper() - Constructor for class org.cloudbus.cloudsim.examples.power.Helper


HISTORY\_LENGTH - Static variable in class org.cloudbus.cloudsim.power.PowerVm
:   The Constant HISTORY\_LENGTH.

hold(int, long) - Static method in class org.cloudbus.cloudsim.core.CloudSim
:   Used to hold an entity for some time.

HOLD\_DONE - Static variable in class org.cloudbus.cloudsim.core.SimEvent


HOLDING - Static variable in class org.cloudbus.cloudsim.core.SimEntity
:   The Constant HOLDING.

HorizontalClustering - Class in org.workflowsim.clustering
:   HorizontalClustering merges task at the same horizontal level

HorizontalClustering(int, int) - Constructor for class org.workflowsim.clustering.HorizontalClustering
:   Initialize a HorizontalClustering Either clusterNum or clusterSize should
    be set

HorizontalDistanceBalancing - Class in org.workflowsim.clustering.balancing.methods
:   HorizontalDistanceBalancing is a method that merges tasks based on distance metric

HorizontalDistanceBalancing(Map, Map, int) - Constructor for class org.workflowsim.clustering.balancing.methods.HorizontalDistanceBalancing
:   Initialize a HorizontalDistanceBalancing object

HorizontalImpactBalancing - Class in org.workflowsim.clustering.balancing.methods
:   HorizontalImpactBalancing is a method that merges tasks that have similar impact
    factors.

HorizontalImpactBalancing(Map, Map, int) - Constructor for class org.workflowsim.clustering.balancing.methods.HorizontalImpactBalancing
:   Initialize a HorizontalImpactBalancing object

HorizontalRandomClustering - Class in org.workflowsim.clustering.balancing.methods
:   HorizontalRuntimeBalancing is a method that merges task so as to balance job runtime

HorizontalRandomClustering(Map, Map, int) - Constructor for class org.workflowsim.clustering.balancing.methods.HorizontalRandomClustering
:   Initialize a HorizontalRuntimeBalancing object

HorizontalRuntimeBalancing - Class in org.workflowsim.clustering.balancing.methods
:   HorizontalRuntimeBalancing is a method that merges task so as to balance job runtime

HorizontalRuntimeBalancing(Map, Map, int) - Constructor for class org.workflowsim.clustering.balancing.methods.HorizontalRuntimeBalancing
:   Initialize a HorizontalRuntimeBalancing object

HorizontalRuntimeVariance - Class in org.workflowsim.clustering.balancing.metrics
:   HorizontalRuntimeVariance is the standard deviation of the runtime

HorizontalRuntimeVariance() - Constructor for class org.workflowsim.clustering.balancing.metrics.HorizontalRuntimeVariance


Host - Class in org.cloudbus.cloudsim
:   Host executes actions related to management of virtual machines (e.g., creation and destruction).

Host(int, RamProvisioner, BwProvisioner, long, List<? extends Pe>, VmScheduler) - Constructor for class org.cloudbus.cloudsim.Host
:   Instantiates a new host.

HOST\_BW - Static variable in class org.cloudbus.cloudsim.examples.power.Constants


HOST\_MIPS - Static variable in class org.cloudbus.cloudsim.examples.power.Constants


HOST\_PES - Static variable in class org.cloudbus.cloudsim.examples.power.Constants


HOST\_PEs - Static variable in class org.cloudbus.cloudsim.network.datacenter.NetworkConstants


HOST\_POWER - Static variable in class org.cloudbus.cloudsim.examples.power.Constants


HOST\_RAM - Static variable in class org.cloudbus.cloudsim.examples.power.Constants


HOST\_STORAGE - Static variable in class org.cloudbus.cloudsim.examples.power.Constants


HOST\_TYPES - Static variable in class org.cloudbus.cloudsim.examples.power.Constants


HostDynamicWorkload - Class in org.cloudbus.cloudsim
:   The class of a host supporting dynamic workloads and performance degradation.

HostDynamicWorkload(int, RamProvisioner, BwProvisioner, long, List<? extends Pe>, VmScheduler) - Constructor for class org.cloudbus.cloudsim.HostDynamicWorkload
:   Instantiates a new host.

hostList - Static variable in class org.cloudbus.cloudsim.examples.power.RunnerAbstract
:   The host list.

HostList - Class in org.cloudbus.cloudsim.lists
:   HostList is a collection of operations on lists of hosts.

HostList() - Constructor for class org.cloudbus.cloudsim.lists.HostList


hostlist - Variable in class org.cloudbus.cloudsim.network.datacenter.Switch


HostPacket - Class in org.cloudbus.cloudsim.network.datacenter
:   HostPacket represents the packet that travels through the virtual network with a Host.

HostPacket(int, int, double, double, double, int, int) - Constructor for class org.cloudbus.cloudsim.network.datacenter.HostPacket


HostStateHistoryEntry - Class in org.cloudbus.cloudsim
:   The Class HostStateHistoryEntry.

HostStateHistoryEntry(double, double, double, boolean) - Constructor for class org.cloudbus.cloudsim.HostStateHistoryEntry
:   Instantiates a new vm mips allocation history entry.

HostToSwitchid - Variable in class org.cloudbus.cloudsim.network.datacenter.NetworkDatacenter

A B C D E F G H I J L M N O P Q R S T U V W Z

- Overview
- Package
- Class
- Use
- Tree
- Deprecated
- Index
- Help

- Prev Letter
- Next Letter

- Frames
- No Frames

- All Classes
